# Controlling and probing heat generation in an optical heater system

**DOI:** 10.1515/nanoph-2021-0604

**Published:** 2022-01-27

**Authors:** Hairegu Tuxun, Zefeng Cai, Min Ji, Baobao Zhang, Chengyun Zhang, Jinping Li, Xudong Yu, Zhengkun Fu, Zhenglong Zhang, Hairong Zheng

**Affiliations:** School of Physics and Information Technology, Shaanxi Normal University, Xi’an, 710119, China; State Key Laboratory of Quantum Optics and Quantum Optics Devices, Shanxi University, Taiyuan, 030006, China

**Keywords:** optical thermometry, plasmonic optical heater, rare-earth doped microrod, silver nano-islands

## Abstract

Understanding how plasmonic nanostructures generate heat upon exposure to light, and thus increase the local temperature of the surrounding medium is important for many applications. Reliable temperature manipulation requires analyzing the local temperature distribution as a function of laser density. In this work, an optical heating system containing silver nano-islands (Ag NIs) is designed to enable heat generation at the micro/nanometer scale and the local temperature can reach 1458 K. The heat generation by Ag NIs exposed to near-IR laser light, and the temperature distribution, are detected *in situ* via the fluorescence intensity ratio technique. It was found that the temperature of the system can be controlled by changing the excitation power. Furthermore, the temperature-dependent UCL of a single Y_2_O_3_:Yb^3+^/Er^3+^ microrod is studied by taking advantage of the controllable local temperature in the optical heating system. It was found that the color of the upconversion luminescence can be tuned by managing the local temperature, and conversely, the local temperature at the optical heater can be monitored by observing the color change of the rare-earth microrod. The real-time manipulation of plasmonic heating offers an opportunity to control outcomes of thermo-plasmonic effects, which then enables a myriad of practical applications.

## 1 Introduction

Precise temperature control at the micro/nanoscale is a significant challenge in nanotechnology across physics, chemistry, and biology [1]. Plasmonic metal nanostructures, which support localized surface plasmons, can be designed to act as effective light-controllable heat sources, which provide very fast heating rates [2], [3], [4], [5], [6], [7], [8], [9]. This has led to a wide range of emerging applications, including photothermal (PT) imaging [10], [11], [12], solar steam generation [13], PT cancer therapy [14], [15], [16], [17], plasmon-mediated photocatalysis [18, 19], plasmon-assisted chemical vapor deposition [20], and heat-assisted magnetic recording [21]. Importantly, most of these applications not only require a fast heating rate but also localized heat generation, high temperature, and large temperature gradients at the micro/nanoscale. Therefore, to realize the full potential of thermo-plasmonics, there is a need for detailed understanding of how heat generation is affected by the irradiation power density applied to the plasmonic micro/nanoheater.

Experimental measurement of temperature, especially of temperature distributions with large gradients, at the micro/nanoscale is challenging. To date, the most commonly used techniques for characterizing plasmonic heaters include thermal cameras [22], scanning thermal microscopy (SThM) [23, 24], Raman scattering [25], and temperature-dependent refractive index change of the surrounding medium [26]. However, each of these has limitations. It is difficult to achieve spatial resolution smaller than ∼2 μm with thermal cameras. The SThM and Raman-scattering based techniques can resolve the heating effects of individual nanoparticles, but require slow scanning to produce the thermal image. Techniques based on thermally-induced variation of the refractive index generally have high temperature sensitivity, but require special sample treatment. Moreover, most of these approaches focus only on steady-state plasmonic heating, while the problem of evaluating fast heating and high temperatures with large temperature gradients remains unresolved. Therefore, it is important to develop a micro/nanothermometer capable of precisely controlling and measuring fast heating, high temperatures, and steep temperature gradients—as typically induced by plasmonic nanostructures. This remains a grand challenge.

The fluorescence intensity ratio (FIR) technique based on trivalent lanthanide (Ln^3+^) doped materials is ideal for the real-time probing of local temperature at the micro/nanometer scale because it can provide rapid response with high accuracy and high spatial resolution. In particular, the Er^3+^/Yb^3+^ codoped system is employed for optical temperature measurement. Thermally-coupled levels require an energy gap, ∆*E*, of 200–2000 cm^−1^; the closely spaced ^4^S_3/2_/^2^H_11/2_ levels of the Er^3+^ activator make a good candidate, and the large absorption cross-section of the Yb^3+^ ion at 980 nm helps achieve strong upconversion (UC) fluorescence emission [27, 28].

In this work, a method for designing an optical heater system at the micro/nanoscale, based on silver nano-islands (Ag NIs), is presented. The local temperature is investigated by analyzing the integrated intensity ratio of emissions from Er^3+^ (^2^H_11/2_ to ^4^I_15/2_ and ^4^S_3/2_ to ^4^I_15/2_ transitions) and local temperatures of up to 1458 K were achieved. It is suggested that Ag NIs can act as a light-triggered micro/nanoheater to rapidly generate high local temperatures. Because the sample environmental temperature directly influences the nonradiative transition rates, the local heating results in changes of spectral characteristics. Since it is difficult to produce large temperature gradients at the micro/nanometer scale with traditional heat sources, the temperature-dependent upconversion luminescence (UCL) of a single Y_2_O_3_:Yb^3+^/Er^3+^ microrod is further studied by taking advantage of the ability to control the local temperature at the optical heater via varying the illumination power. Distinct changes from red to green are observed, owing to temperature changes caused by changing the excitation power. In addition, distinct green and red regions were present when a single microrod was excited at one end. The green appeared in the vicinity of the excitation point, while the opposite end was red. This result indicates that there was a large temperature gradient in the optical heater. Our investigation may help to elucidate heat generation in plasmonic nanostructures, and potential applications of the combination of microrods and optical heaters include in the anti-counterfeiting field.

## 2 Materials and methods

### 2.1 Sample preparation

NaYF_4_: Yb^3+^/Er^3+^ microrods were prepared by a hydrothermal method [29]. Y_2_O_3_, Yb_2_O_3_, and Er_2_O_3_ were supplied by Aladdin Chemical Reagent Company. NH_4_F and sodium citrate were purchased from Sinopharm Chemical Reagent Co., Ltd of China. All chemicals were of analytical grade and used without further purification. Deionized water was used in all experiments.

The procedure for preparing NaYF_4_:Yb^3+^/Er^3+^ microrods via the hydrothermal method was as follows. First, Y_2_O_3_, Yb_2_O_3_, and Er_2_O_3_ powders were dissolved in dilute nitrate solution and the residual nitrate was removed by heating and evaporation. The clear solution of Ln(NO_3_)_3_ (Ln = Y, Yb, Er) is obtained, which will be used in the later reaction. Subsequently, 12.5 mL of aqueous solution containing 0.4 g EDTA and 1.05 mL of NaOH aqueous solution (5.0 M) were mixed under stirring until the solution became clear. After that, 5 mL of Ln(NO_3_)_3_ (Ln = Y, Yb, Er) aqueous solution (0.2 M), 8 mL of NH_4_F (2.0 M) aqueous solution and 7 mL dilute hydrochloric acid (1 M) were added to the mixture. After additional agitation for 90 min with magnetic stirring, the mixed solution was transferred into a teflon-lined autoclave, and heated at 200 °C for 24 h. The final products were collected by centrifugation, and then washed with deionized water and ethanol for several times. Finally, the obtained NaYF_4_:Yb^3+^/Er^3+^ samples were dried at 60 °C for 12 h.

Ag NIs coated Y_2_O_3_:Er^3+^/Yb^3+^ microrods were obtained from NaYF_4_:Er^3+^/Yb^3+^ microrods through a PT catalysis reaction caused by a surface plasmon effect [30]. In the first step, isolated NaYF_4_:Er^3+^/Yb^3+^ microrods were distributed on pre-cleaned glass surfaces. Subsequently, a 20 nm thick silver film was evaporated onto the treated surface under a high vacuum environment. By applying proper laser irradiation at a wavelength of 980 nm, Y_2_O_3_:Er^3+^/Yb^3+^ coated with Ag NIs-referred to as “optical heaters” in this study-were obtained. Figures S2 to S9 in the supporting information show detailed characterization of the optical heaters.

### 2.2 Characterization

To characterize the samples, scanning electron microscopy (SEM) was conducted with an FEI – Nova Nano-SEM 450 at 10 kV. Atomic force microscopy images were obtained with a Bruker-JPK NanoWizard ULTRA. X-ray diffraction was performed with a Rigaku D/Max2550VB+/PC diffractometer at a scanning rate of 7°min^−1^ with Cu K*α* (40 kV, 40 mA) radiation (*λ* = 0.15406 nm) and a graphite monochromator. High-angle annular dark-field, selected area electron diffraction, and energy-dispersive x-ray images were obtained with an FEI Titan Cubed Themis G2 300 microscope operated at 300 kV and equipped with a probe aberration corrector and monochromator. The optical measurements were conducted with a confocal UC fluorescence microscopy system. During the spectroscopic measurement, samples were excited with a laser (Mira 900-F, Coherent) at 980 nm; the UC fluorescence emission was detected using a spectrometer (SP2750i, PI). The optical images were recorded with a 100× objective (NA = 0.9, Olympus).

### 2.3 Simulations

Numerical simulations were performed by using the finite element method in COMSOL Multiphysics. The models, including morphology parameters of Ag NIs, were constructed based on the experimental system. The dielectric constants of the plasmonic metals were obtained from the data reported by Johnson and Christy [31].

## 3 Results and discussion

**Figure 1:**
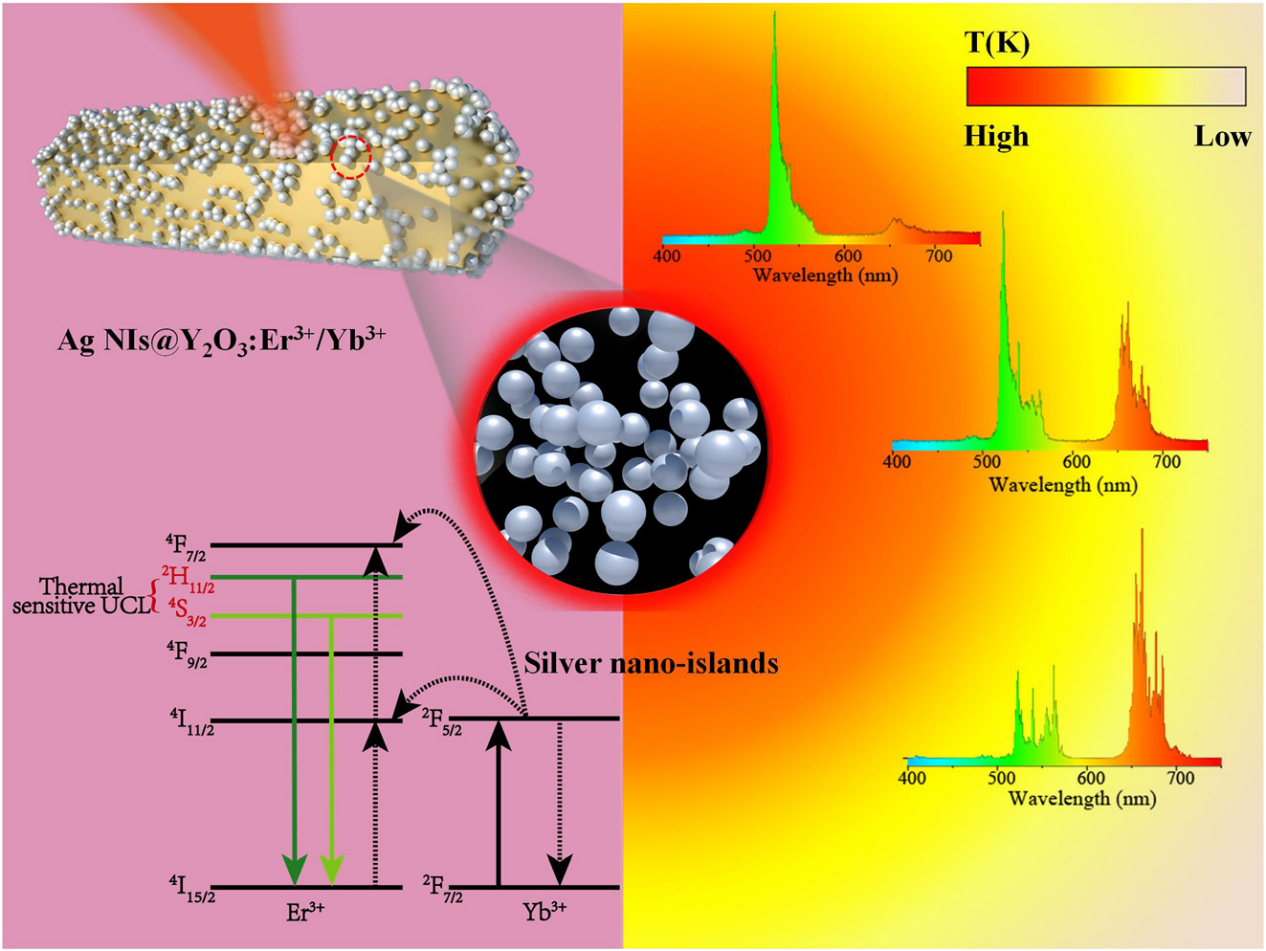
A schematic of temperature monitoring in the optical heating system. The local temperature change is measured by detecting the change in the ratio of the thermally-coupled emissions of Er^3+^ (^2^H_11/2_ → ^4^I_15/2_ and ^4^S_3/2_ → ^4^I_15/2_). The color of the luminescent UC of the microrod can be tuned by controlling the temperature of the optical heating system. With increasing light irradiation power, the luminescence emission color gradually changes from red to green.

A schematic illustration of the system used in the current study is shown in Figure 1. The plasmonic Ag NIs, coated onto the surface of the microrods, act as optical heaters; Er^3+^/Yb^3+^ doped Y_2_O_3_ microrods were introduced for temperature detection. Y_2_O_3_ was selected as the host material because of its high chemical stability and high melting point [32]. The luminescence energy levels of Er^3+^ (^2^H_11/2_ → ^4^I_11/2_, and ^4^S_3/2_ → ^4^I_15/2_) are thermally coupled; thus, the FIR can be used as a primary thermometer. The optical thermometer Y_2_O_3_:Er^3+^/Yb^3+^ has good contact with Ag NIs, and Ag NIs are uniformly dispersed on the surface of the Y_2_O_3_:Er^3+^/Yb^3+^. Good contact is beneficial to the heat transfer of Ag NIs to Y_2_O_3_:Er^3+^/Yb^3+^, thus achieving the thermal equilibrium quickly. Meanwhile the optical thermometer allows us to detect the local temperature *in situ* during the light illumination. As local temperature changes are induced by the optical heater, the microrod exhibits clear color changes from red to yellow to green. Different colors in Figure 1 represent the temperature changes of the microrod in the thermal gradient.

To clarify how the PT capacity of the optical heater varies with the illuminating power, theoretical simulations were performed as shown Figure S10(a) and (b).

### 3.1 Local temperature detection for optical heater system

**Figure 2:**
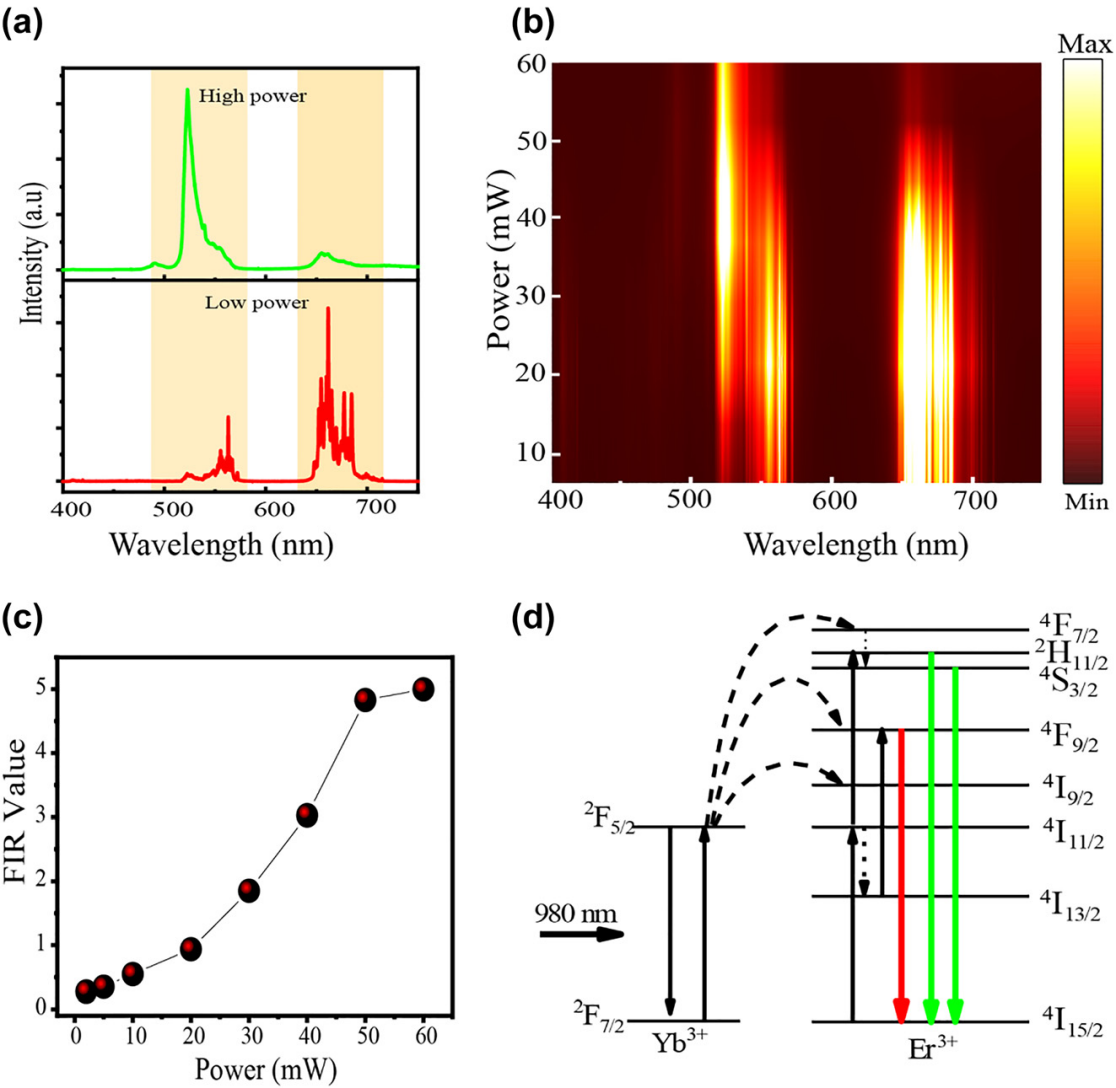
Power dependence of upconversion emission spectra and FIR value for the Y_2_O_3_:Er^3+^/Yb^3+^ with optical heater. (a) UC emissions (at low and high power excitation) from Y_2_O_3_:Er^3+^/Yb^3+^ microrod coated with optical heater Ag NIs; (b) excitation-power dependence of UC emission from Y_2_O_3_:Er^3+^/Yb^3+^ microrod coated with optical heater Ag NIs; (c) dependence of FIR value, *I*_523_/*I*_555_, on the excitation power; (d) energy level diagram and corresponding transitions of Yb^3+^ and Er^3+^.

To study the local temperature produced by NIR irradiation at different laser power densities, UC emission spectra from Y_2_O_3_:Er^3+^/Yb^3+^ microrods coated with Ag NIs were measured. Figure 2(a) presents the UC emission from the Y_2_O_3_:Er^3+^/Yb^3+^ microrods at low and high power excitation. The green emission (475–580 nm) corresponds to ^2^H_11/2_, ^4^S_3/2_ → ^4^I_15/2_ transitions, while the red emission (630–720 nm) is attributed to ^4^F_9/2_ → ^4^I_15/2_ transitions of the Er^3+^ ion. As shown in Figure 2(b), upon increasing the excitation power, the intensity of the red emission bands decreased while that of the green emission increased. In addition, the integrated FIR of ^2^H_11/2_ → ^4^I_15/2_ to ^4^S_3/2_ → ^4^I_15/2_ increased from 0.35 to 5.00 as the excitation power increased from 5 to 60 mW, as shown in Figure 2(c). This indicates that the temperature of the microrod has increased, because increasing the temperature of the microrod could induce a thermally-driven population increase at the ^2^H_11/2_ level, at the expense of the population of the lower-energy ^4^S_3/2_ level. According to Boltzmann’s distribution, the FIR [33, 34] is:

(1) $$I_{523}/I_{555}=C\;exp\left(-\frac{\Delta E}{KT}\right)$$

where *C* is a proportionality constant, and *I*_523_ and *I*_555_ are integrated intensities of the ^2^H_11/2_ → ^4^I_15/2_ and ^4^S_3/2_ → ^4^I_15/2_ transitions, respectively. ∆*E* is the energy gap between the two excited states, *k* is the Boltzmann constant, and *T* is the temperature. The optical micro/nanoheater (Ag NIs) and thermometer (Y_2_O_3_:Er^3+^/Yb^3+^ microrods) are close together (Figures S1 and S5), so the local temperature *T* of the system is readily determined from the FIR.

**Figure 3:**
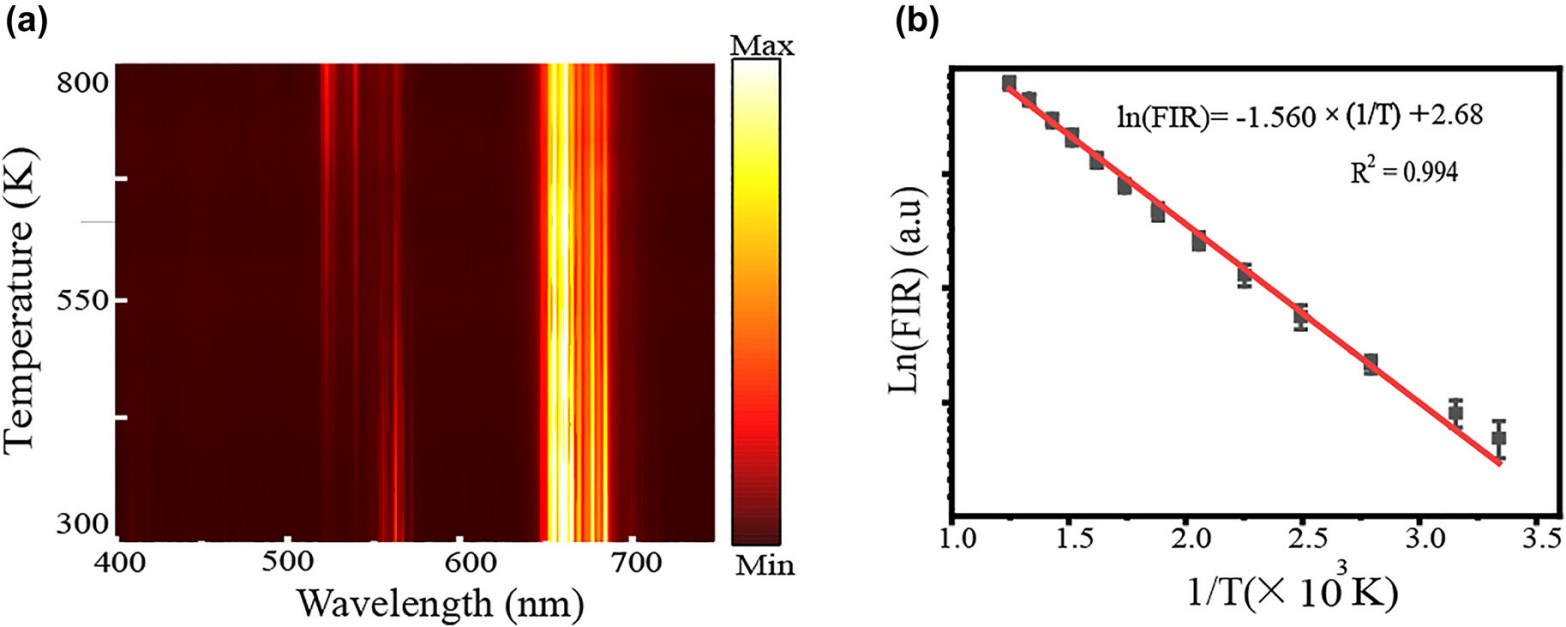
Calibration curve to built the relationship between UCL intensity and temperature. (a) Temperature-dependent UC emission of Y_2_O_3_:Er^3+^/Yb^3+^ microrod obtained on a conventional heating stage; (b) FIR as a function of temperature (dots) and fitting result (solid line).

Since the FIR technique is based on thermally-coupled levels, it is necessary to create a calibration curve to build the relationship between UCL intensity and temperature. Figure 3(a) is the temperature-dependent UC emission of bare Y_2_O_3_:Er^3+^/Yb^3+^ microrods (without Ag NIs), from 300 to 802 K, obtained with conventional heating. During the luminescence emission measurement, a low excitation power (*P* = 15 mW) was employed to avoid laser-induced thermal effects. As the temperature increases from 300 to 802 K, the FIR increases from 0.08 to 2.20. This is because the electrons in the ^4^S_3/2_ state can migrate to the ^2^H_11/2_ state via a thermalization process, leading to increased intensity for the ^2^H_11/2_ → ^4^I_15/2_ transition and decreased intensity for the ^4^S_3/2_→ ^4^I_15/2_ transition, which results in an increase in FIR. A similar increase in FIR was found when optical heaters were employed with increasing pump powers, and was attributed to an increase in temperature with increasing pump power. By fitting the experimental data in Figure 3(b), the following relationship between FIR and temperature for conventional heating was obtained: Ln(FIR) = −1.560 × (1/*T*) + 2.68. The value of ∆*E* was 1084 cm^−1^, which is similar to the value obtained for the sample with the optical heater. Generally speaking, thermally-coupled energy levels with small energy gap are more suitable for detection of lower-temperature regions, while coupled energy levels with a larger energy gap may show “thermal decoupling” at low temperature, but show good temperature-sensing performance at high temperatures [35, 36]. Therefore, Er^3+^/Yb^3+^ codoped Y_2_O_3_ microcrystals are expected to be effective thermometers at higher temperatures.

As previously reported for optical temperature sensing, the temperature of the optical heater can easily be evaluated from Eq. (2) [37],

(2) $$T=\frac{1}{\text{ln }C-\text{ln }\left(\frac{I_{523}}{I_{555}}\right)}$$

**Figure 4:**
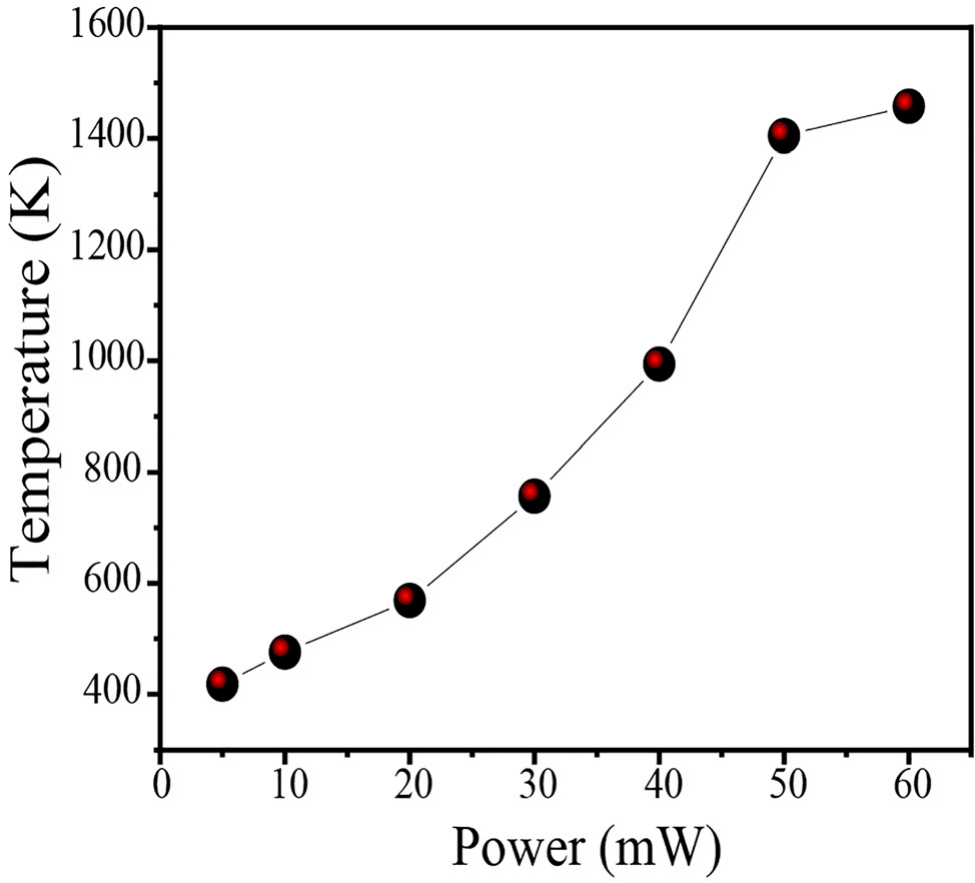
The power-dependent temperature at the optical heater, calculated from the FIR value (*I*_523_/*I*_555_).

Here, *I*_523_ and *I*_555_ are the integrated intensities of the ^2^H_11/2_ → ^4^I_15/2_ and ^4^S_3/2_ → ^4^I_15/2_ transitions, respectively. By fitting the experimental data in Figure 3(b) to Eq. (2), the values of temperature represented by the FIRs are obtained, as shown by the solid line in Figure 4. As the irradiation laser power increases from 5 to 60 mW, the temperature increases monotonically from 418 to 1458 K.

Under excitation with an NIR laser, the UCL materials cannot only convert the absorbed energy to UC emission, but could also convert some of the absorbed energy into heat, through nonradiative processes. If present, this thermal effect would also exist for the bare Y_2_O_3_:Er^3+^/Yb^3+^ microrod (without Ag NIs). To confirm that the thermal effects induced by laser irradiation in the optical heater system mainly arise from the thermoplasmonics of the micro/nanoheater (Ag NIs), the variation of the UC emission spectra and FIR with excitation power for a bare microrod was also investigated, and the result is shown in Figure S11. As the radiation power density increased from 5 to 60 mW, the total intensity increased gradually, but no significant change was detected in the emission spectra. The FIR value increased from 0.07 to 0.23, which corresponds to a temperature change from 293 to 376 K. Table 1 lists the temperatures obtained from the FIR of the Er^3+^ ion in Y_2_O_3_:Er^3+^/Yb^3+^, with and without Ag NIs. These results demonstrate that the thermal heating caused by the laser on the UC microrod is very small, and its influence can be neglected when studying this optical heater system. The Ag NIs coated onto the microrod is responsible for the vast majority of the heating when excited at 980 nm.

**Table 1:** Temperatures obtained from FIR measurement at different excitation powers.

Y_2_O_3_:Er^3+^/Yb^3+^			Y_2_O_3_:Er^3+^/Yb^3+^ coated with Ag NIs (optical heater)	
Irradiation power (mW)	FIR value	Temperature (K)	FIR value	Temperature (K)
5	0.07	293	0.35	418
10	0.08	300	0.55	476
20	0.12	325	0.94	569
30	0.15	341	1.85	757
40	0.19	359	3.03	994
50	0.21	368	4.83	1405
60	0.23	376	5.00	1458

### 3.2 Temperature-dependent UCL of a single Y_2_O_3_:Er^3+^/Yb^3+^ microrod

In the process of UCL emission, nonradiative relaxations are involved, and their transition rates usually depend on the surrounding temperature, resulting in a temperature-dependent change in luminescence properties. Due to the limitation of experimental conditions, it is difficult to study the temperature-dependent luminescence of a single particle at high temperature. In addition, traditional heat sources are hard to produce large temperature gradient in the micro/nanometer scale. Therefore, studies of the temperature-dependent UCL will be helpful to further our understanding of the UCL process, especially for the development of temperature detection at the micro/nanoscale.

**Figure 5:**
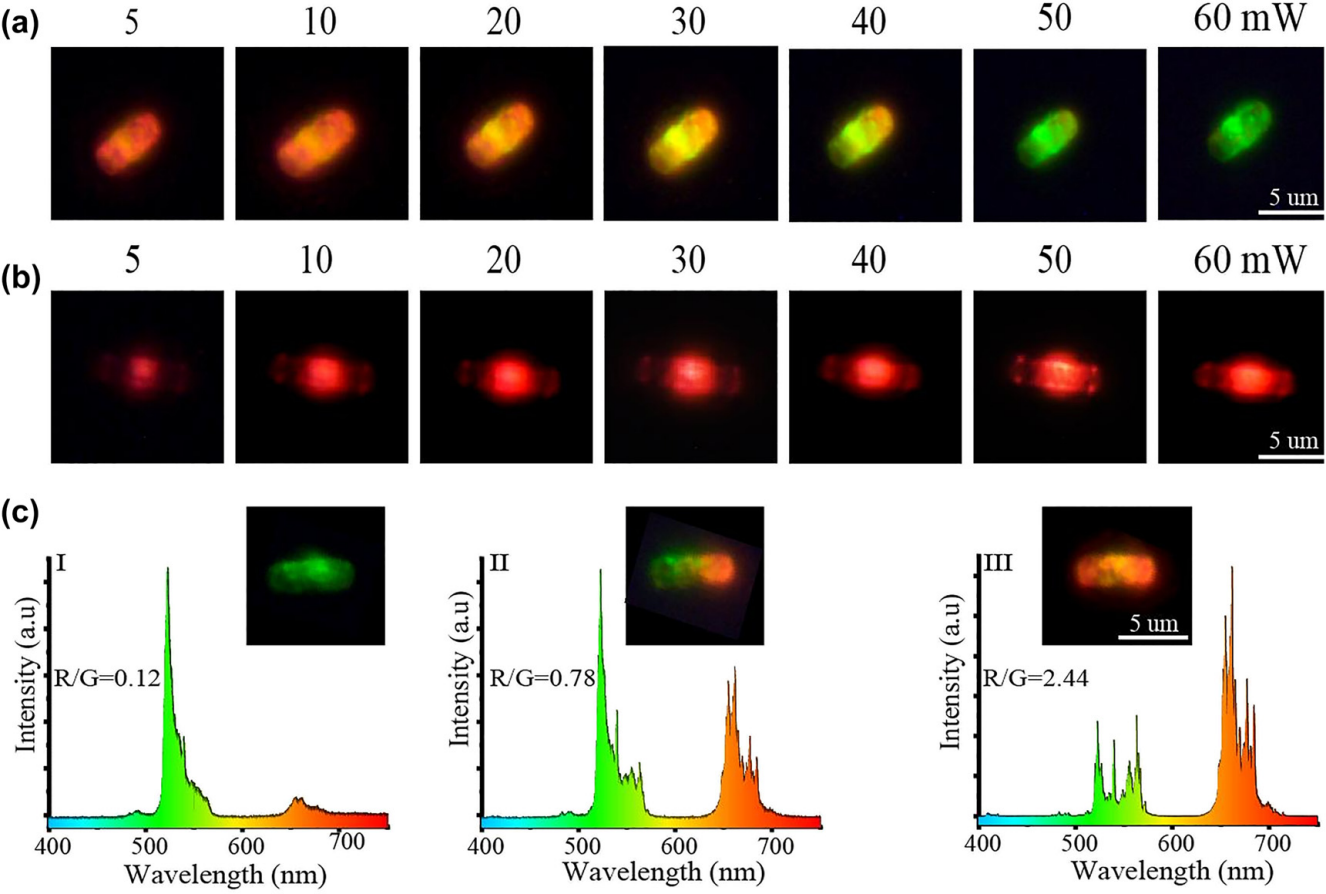
Luminescence emission patterns and spectra for the Y_2_O_3_:Er^3+^/Yb^3+^ with and without optical heater. (a) Luminescence patterns of a single Y_2_O_3_:Er^3+^/Yb^3+^ microrod coated with optical heaters and measured at different excitation powers; (b) luminescence patterns of a single bare Y_2_O_3_:Er^3+^/Yb^3+^ microrod, measured at different excitation powers; (c) UCL pattern and emission spectra of a Y_2_O_3_:Er^3+^/Yb^3+^ microrod coated with Ag NIs, excited at the center (I) end (II) and (III) side edge of the microrod.

Taking advantage of the optical heater can provide controllable high temperature in a very short time with different power illumination, we further investigated the temperature-dependent UCL of a single Y_2_O_3_:Yb^3+^/Er^3+^ microrod coated by optical heaters. It was observed that the emission color changed continuously from red to yellow to green as the power density increased from 5 to 60 mW, demonstrating that the emission could be tuned by changing the excitation power density (Figure 5(a)).

To verify the possible influence of excitation light on the local temperature change, the power-dependent UC emission spectrum of a bare Y_2_O_3_:Yb^3+^/Er^3+^ microrod was also measured (Figure S11(a)). No significant change was detected except for a gradual increase in total intensity with increasing excitation power density. The power-dependent luminescence patterns for a single bare Y_2_O_3_:Yb^3+^/Er^3+^ microrod are shown in Figure 5(b). Combining these results implies that the tunable emission color results mainly from changes in the local temperature. Under irradiation from an NIR laser, the Ag NIs coated onto the microrod constantly convert optical energy into plasmonic heat at the micro/nanometer scale; the converted thermal energy can be transferred to the sample immediately. Increasing the NIR laser power induces a temperature rise in the optical heater, which leads to the luminescence color changes. Remarkable enhancement in green emission from the ^2^H_11/2_ → ^4^I_15/2_ transition and reduction in red emission from the ^4^F_9/2_ → ^4^I_15/2_ transition were detected, and the emission color showed a continuous evolution from red to yellow and green (Figure 5(a)).

We also carefully investigated the UCL pattern and spectra (Figure 5(c)) when a single microrod was excited at different positions. Figure S12 presents a schematic illustration of the sample when it is excited at the center, side edge, and one end. Figure 5(c) shows that the sample exhibits green and yellow emission when it is excited at the center or at the side edge. More interestingly, separate green and red regions form when the microrod is excited at one end. The green area is around the excitation point, while the red appears at the opposite end. This phenomenon could originate from a gradual change of local non-radiative relaxation probability, corresponding to the temperature gradient along the microrod arising from the plasmonic heating. This type of reversible temperature-sensitive multicolor display in micro/nanomaterials is easily recognized with the naked eye, and shows great promise for developing a more secure anti-counterfeiting method.

## 4 Conclusions

An optical heater comprising plasmonic Ag nano-islands was applied to the surface of microrods, and the thermal effects and local temperature detection with FIR technique were investigated under laser illumination. Upon infrared laser excitation (up to 60 mW), a plasmon-induced temperature change was observed. The local temperature was measured by monitoring the relative intensity of the Er^3+^ UC emission from ^2^H_11/2_, ^4^S_3/2_ to ^4^I_15/2_; the temperature of the system could range from 418 to 1458 K. Y_2_O_3_:Er^3+^/Yb^3+^ microrods coated with the optical heater exhibited clear, power-dependent UCL; the UC emission color gradually altered from red to green as the temperature was altered by the excitation power, without any changes in the crystalline phase structure of the microrods. In addition, because of the temperature gradient in the optical heater, the emission color represents the corresponding change along the longitudinal direction when a single microrod is excited at an edge. Such an optical heating system may enable potential applications of thermal effects and RE ions in anti-counterfeiting and multicolor displays.

## Supplementary Material

Supplementary Material
