# Supplementary material for: Controlling and probing heat generation in an optical heater system
Source: Nanophotonics. 2022 Jan 27;11(5):979–86. doi: 10.1515/nanoph-2021-0604 (PMC11501617; doi:10.1515/nanoph-2021-0604)
Supplement: Supplementary file 1 — Supplementary Material [file j_nanoph-2021-0604_suppl.docx]

**Controlling and probing heat generation in an optical heater system**

Hairegu Tuxun^1^, Zefeng Cai^1^ , Min Ji^1^, Baobao Zhang^1^, Chengyun Zhang^1^, Jinping Li^1^, Xudong Yu^2^, Zhengkun Fu^1*^, Zhenglong Zhang^1^, Hairong Zheng^1*^

*^1^School of Physics and Information Technology, Shaanxi Normal University, 710119，Xi'an, China*

*^2^State Key Laboratory of Quantum Optics and Quantum Optics Devices, Shanxi University,* *Taiyuan, 030006, China*

*Email: [zkfu@snnu.edu.cn](mailto:zkfu@snnu.edu.cn) (Z. K. Fu) and [hrzheng@ snnu.edu.cn](mailto:hrzheng@%20snnu.edu.cn%20)  (H. R. Zheng)

Supporting information -1


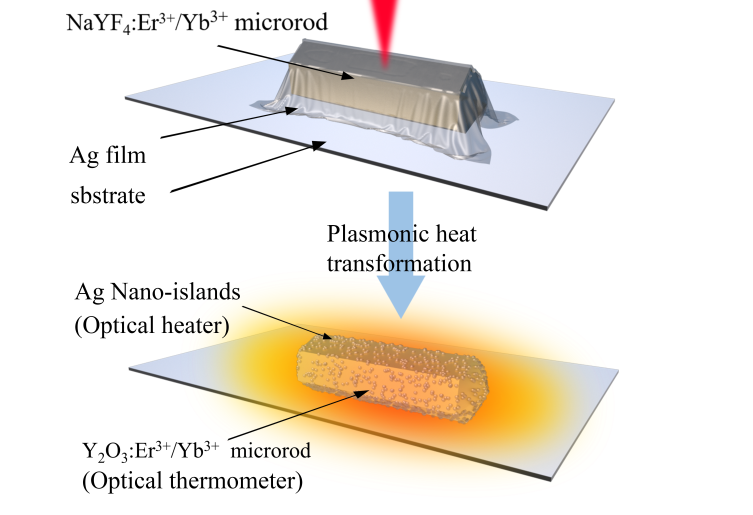


Figure S1. Schematic of the obtained optical heater (Ag NIs) coated optical thermometer Y_2_O_3_:Er^3+^/Yb^3+^ with the assistance of photothermal effect.

Ag NIs coated Y_2_O_3_:Er^3+^/Yb^3+^ microrods were obtained from NaYF_4_:Er^3+^/Yb^3+^ microrods through a PT catalysis reaction caused by a surface plasmon effect [1]. In the first step, isolated NaYF_4_:Er^3+^/Yb^3+^ microrods were distributed on pre-cleaned glass surfaces. Subsequently, a 20 nm thick silver film was evaporated onto the treated surface under a high vacuum environment as shown Figure S1. By applying proper laser irradiation at a wavelength of 980 nm, optical thermometer Y_2_O_3_:Er^3+^/Yb^3+^ coated with Ag NIs-referred to as “optical heaters” in this study-were obtained. Under laser irradiation, the LSPR can be excited and decayed into electron-hole pairs through Landau damping [2]. The hot carriers rapidly transfer energy to the lattice via elastic electron-electron scattering and electron-phonon coupling, and a high lattice temperature occurs over several picoseconds [3]. The heat generated by the Ag NIs is transferred to NaYF_4_, which enables a fast temperature equilibration. When enough thermal energy conveyed to the crystal, reactant NaYF_4_ will rapidly transform into Y_2_O_3_ and finally recrystallize to crystal over tens of milliseconds.

Supporting information -2

The hexagonal NaYF_4_:Er^3+^/Yb^3+^ microrods were synthesized by the hydrothermal method. As shown by scanning electron microscope (SEM) image in Figure S2 (a), the particles are uniform microrods. A lattice spacing of 0.263 nm indicates the hexagonal (101) lattice plane of the microcrystal in Figure S2 (b). The XRD pattern shown in Figure S2 (c) is consistent with the standard peak position of the hexagonal phase of NaYF_4_ (JCPDS file NO.16-0334).


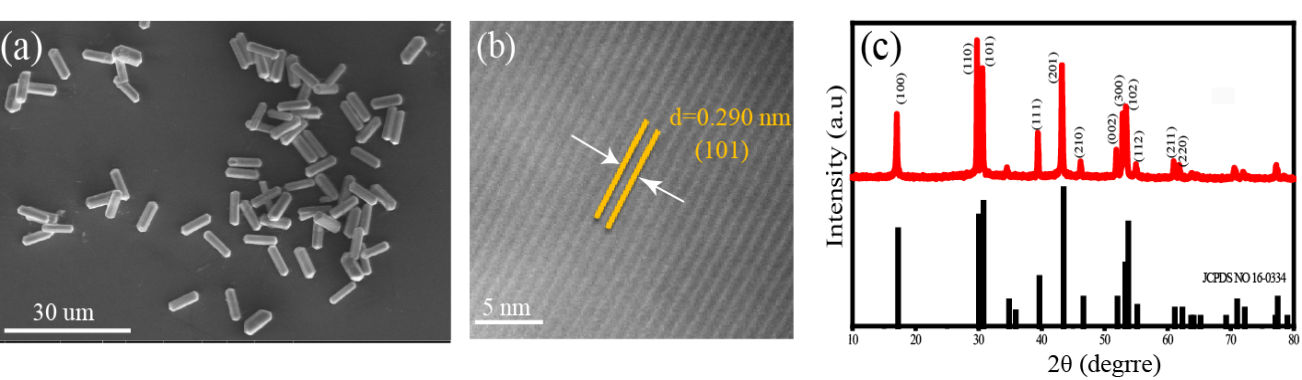


Figure S2. Characterization of NaYF_4_:Er^3+^/Yb^3+^ microrod (a) SEM image; (b) HR-TEM image; (c) XRD pattern.

Supporting information -3

The SEM image and EDX elemental mapping image as shown in Figure S3 (a) and (b), indicate that the Ag film is uniformly deposited on the surface of NaYF_4_:Er^3+^/Yb^3+^ on the glass substrate.


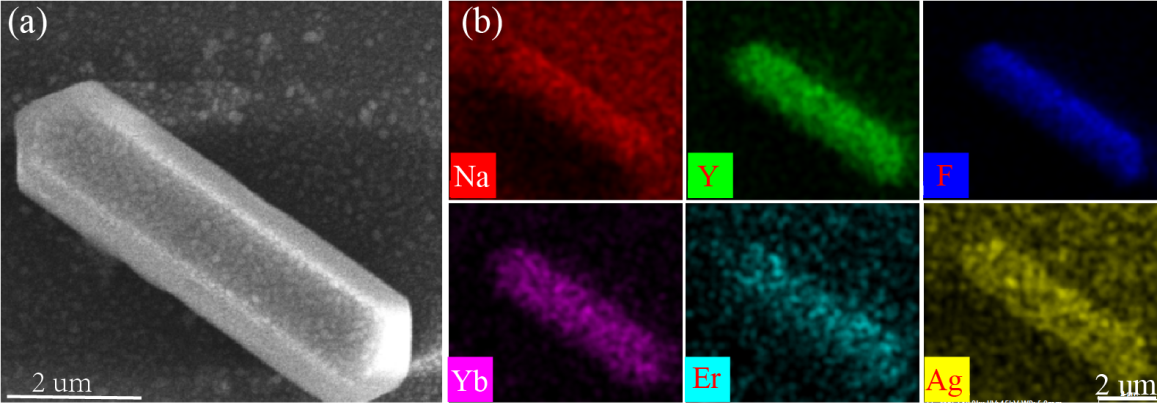


Figure S3. (a) SEM image of the microrod coated with silver film; (b) EDX elemental mapping.

Supporting information -4

Ag NIs coated Y_2_O_3_:Er^3+^/Yb^3+^ microrod is obtained from NaYF_4_:Er^3+^/Yb^3+^ microrod through the photothermal catalysis of surface plasmon effect. As shown in Figure S4, the spectral changes also indicate that the emission arises from Er^3+^/Yb^3+^ doped into Y_2_O_3_.


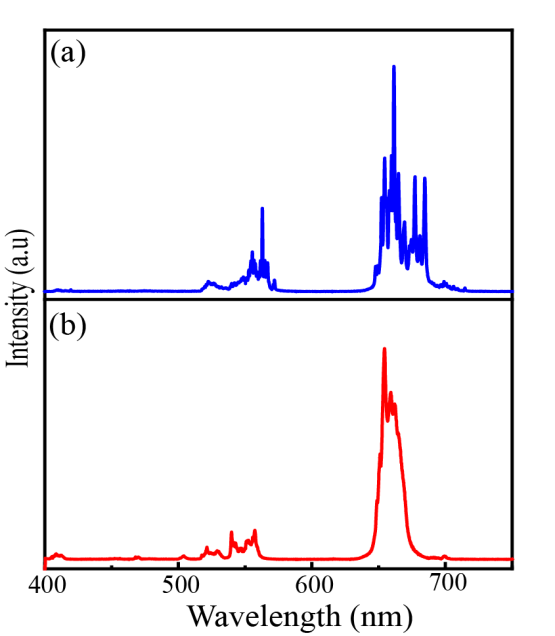


Figure S4. Luminescence spectra of (a) in situ transformed to Y_2_O_3_:Er^3+^/Yb^3+^ with nano-island after laser irradiation with power density of 60 mW. (b) NaYF_4_:Er^3+^/Yb^3+^ with Ag films before using high power irradiated (5 mW).

Supporting information -5

After laser irradiation, the silver film is transformed into silver nano-islands and coated on the surface of Y_2_O_3_:Er^3+^/Yb^3+^ as shown Figure S5 (a). The energy dispersive x-ray (EDX) elemental mapping analysis further proves the transformation from NaYF_4_ to Y_2_O_3_ as shown Figure S5 (b)-(f).


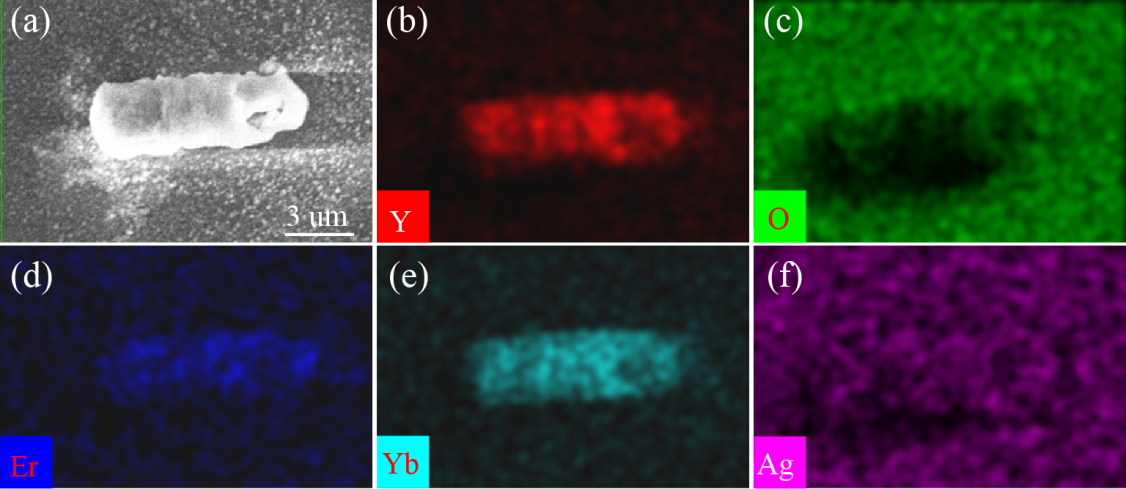


Figure S5. (a) SEM image of the microrod Y_2_O_3_:Er^3+^/Yb^3+^ coated with nano-islands; (b)-(f) EDX elemental mapping.

Supporting information -6

To confirm the transformation, The selected area electron diffraction (SAED) pattern is carried out as shown in Figure S6 (a), which confirms that the resulting particle is single-crystal Y_2_O_3_ with a cubic $\mathrm{Ia}\overline{3}$ structure and lattice parameter $a=10.604A^{^{\circ}}$. The lattice fringes spacing of 0.53 nm fits well with the Y_2_O_3_ faces of (200) as shown in Figure S6 (b). These results indicate that the crystal transformation of NaYF_4_ into crystal Y_2_O_3_ through a PT catalysis reaction caused by a surface plasmon effect.


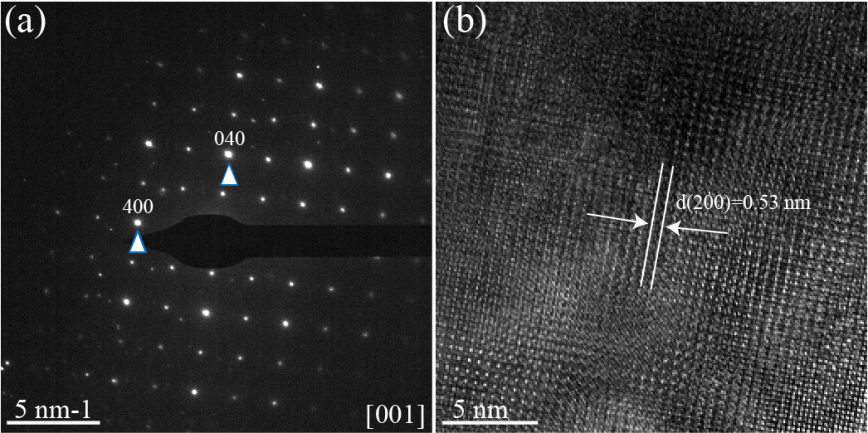


Figure S6. SAED pattern of the Y_2_O_3_:Er^3+^/Yb^3+^ microrod coated with nano-islands is obtained from NaYF_4_:Er^3+^/Yb^3+^ microrod through the photothermal catalysis of surface plasmon effect.

Supporting information -7

From Figure S7 (a) and (b), the average size of the nano-islands is about 100-200 nm.


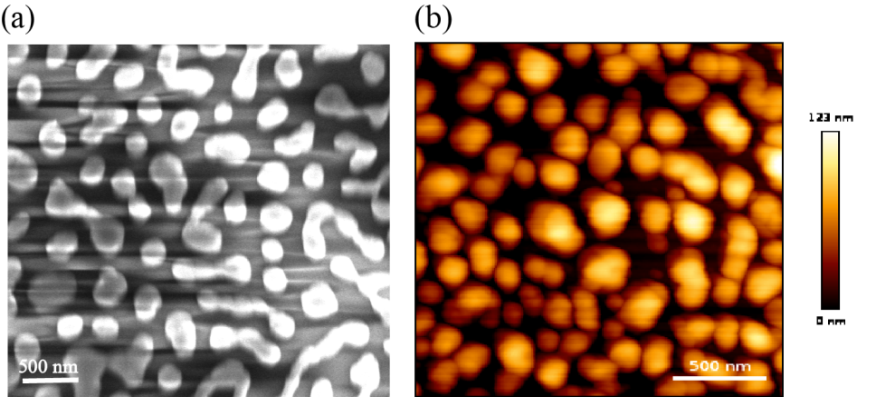


Figure S7. (a) SEM and (b) AFM image for the Ag NIs.

Supporting information -8

Luminescence spectra of the single microrod NaYF_4_:Er^3+^/Yb^3+^ before and after laser irradiation are acquired on a glass substrate without coated by Ag NIs. No changes are observed in the luminescence spectra without coated by Ag NIs even after laser irradiation 30 min (Figure S8), which indicate that the plasmonic thermal effect play an important role for the crystal transformation NaYF_4_ in to Y_2_O_3_.


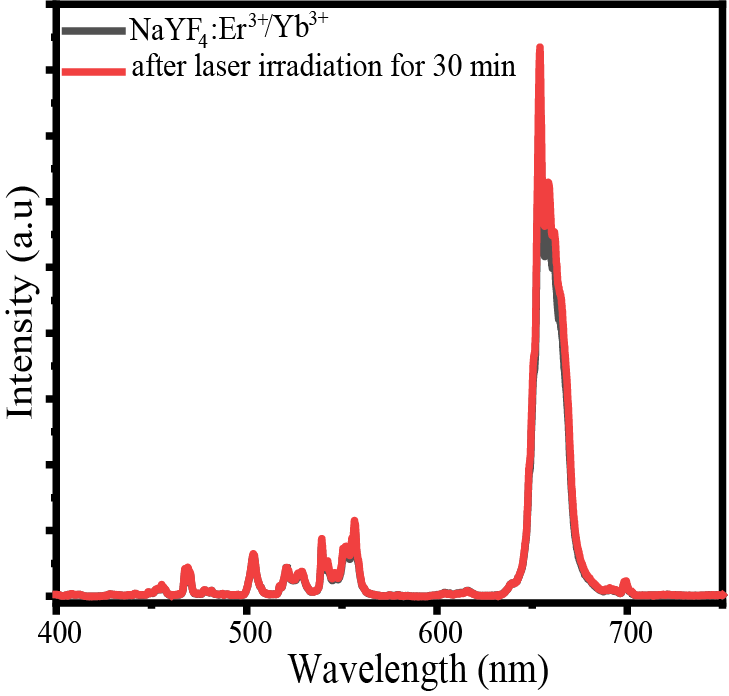


Figure S8. Control experiment, luminescence emission spectra of single NaYF_4_:Er^3+^/Yb^3+^ microrod without coated Ag NIs. Black and red lines are before and after CW laser irradiation (980 nm) for 30 mins, respectively.

Supporting information -9


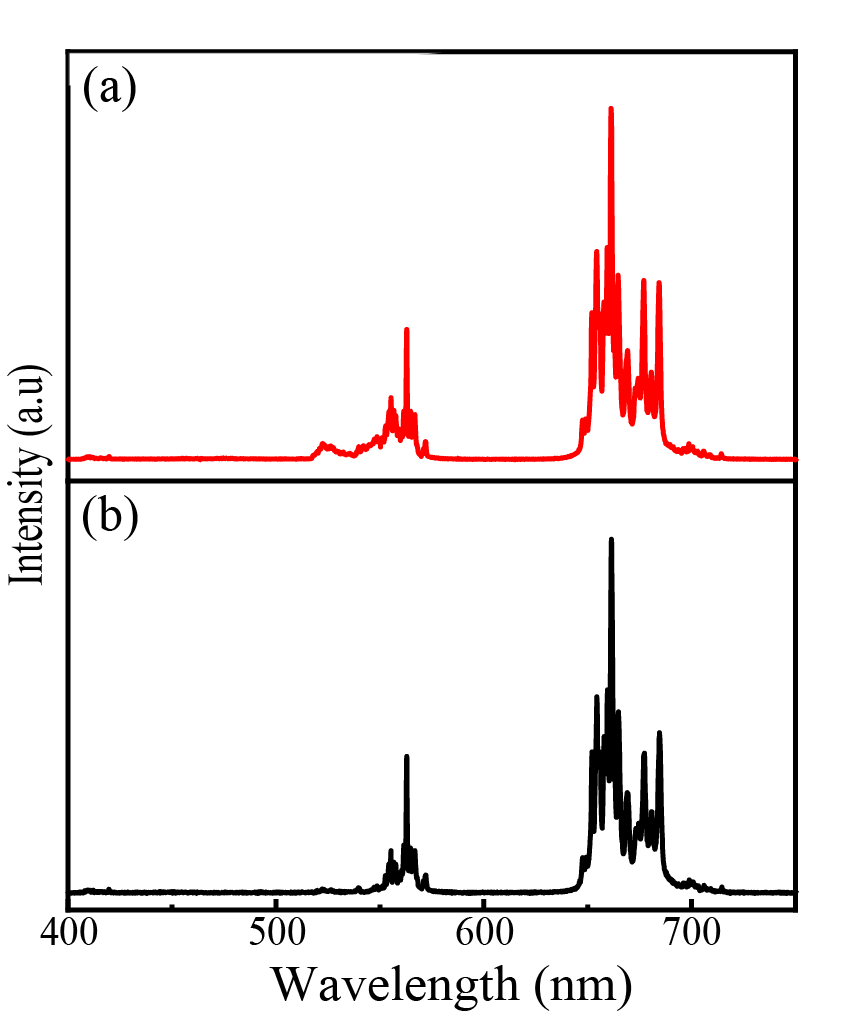


Figure S9. Upconversion emission spectra of the Y_2_O_3_:Er^3+^/Yb^3+^ obtained via (a) Thermal catalytic effect; (b) Conventional thermal annealing at 600℃ temperature for 1 hour.

Supporting information -10

**
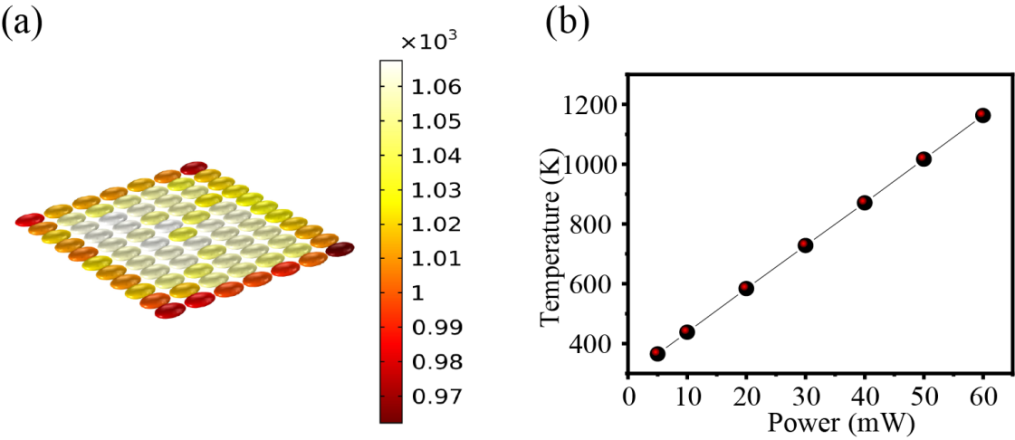
**

Figure S10. (a) Schematic of the temperature distribution in the optical heater composed of Ag NIs arrays; (b) the calculated temperature dependence of optical heater on the excitation power.

To clarify the mechanism of the high photothermal capacity of optical heater varying with the power illuminance, the theoretical simulation is performed as shown Ag NIs array is shown Figure S10 (a). Under proper excitation, the local temperature of the Ag NIs will increase, and the increased temperature is given by the equation of [4]:

$\Delta T=\frac{I\sigma_{\mathrm{abc}}}{4\pi kR}$ (1)

Where $\sigma_{\mathrm{abc}}$ is the absorption cross-section of the Ag NPs, I is the irradiance (optical power per unit area) R is the radius of Ag NIs. The LSPR of Ag NIs can be simply derived for a metallic ellipsoid that is much smaller than the illumination wavelength.

According to equation (1), for a given size and absorption cross section, the local temperature generated by metal nanostructure depends on the power density. Figure S10 (b) demonstrates the power dependence photothermal capacity of an optical heater composed of Ag NIs. Temperature increases with the increase of power density. As the laser power increased from 5 to 60 mW, the temperature increased monotonically from 365 to 1162 K.

Supporting information -11


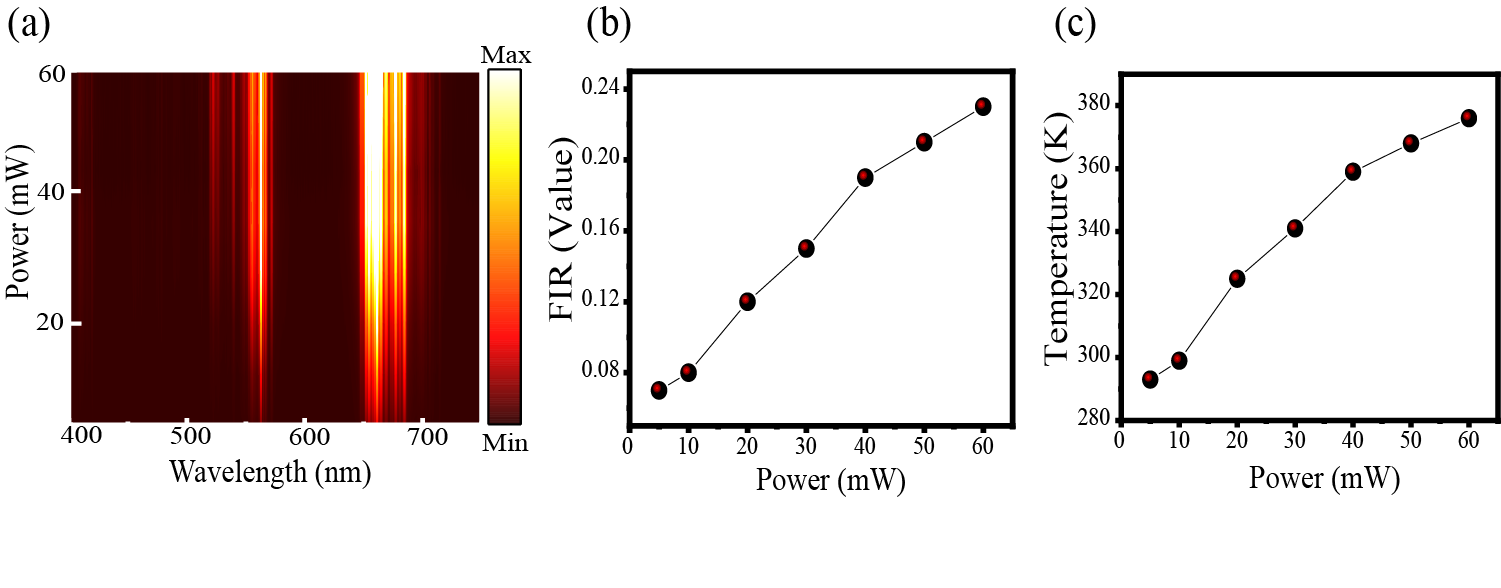


Figure S11. Power dependence (a) luminescence spectra; (b) FIR value (I_523_/I_555_) ; (c) Calculated temperature for the bare Y_2_O_3_:Yb^3+^/Er^3+^ microrod.

Supporting information -13


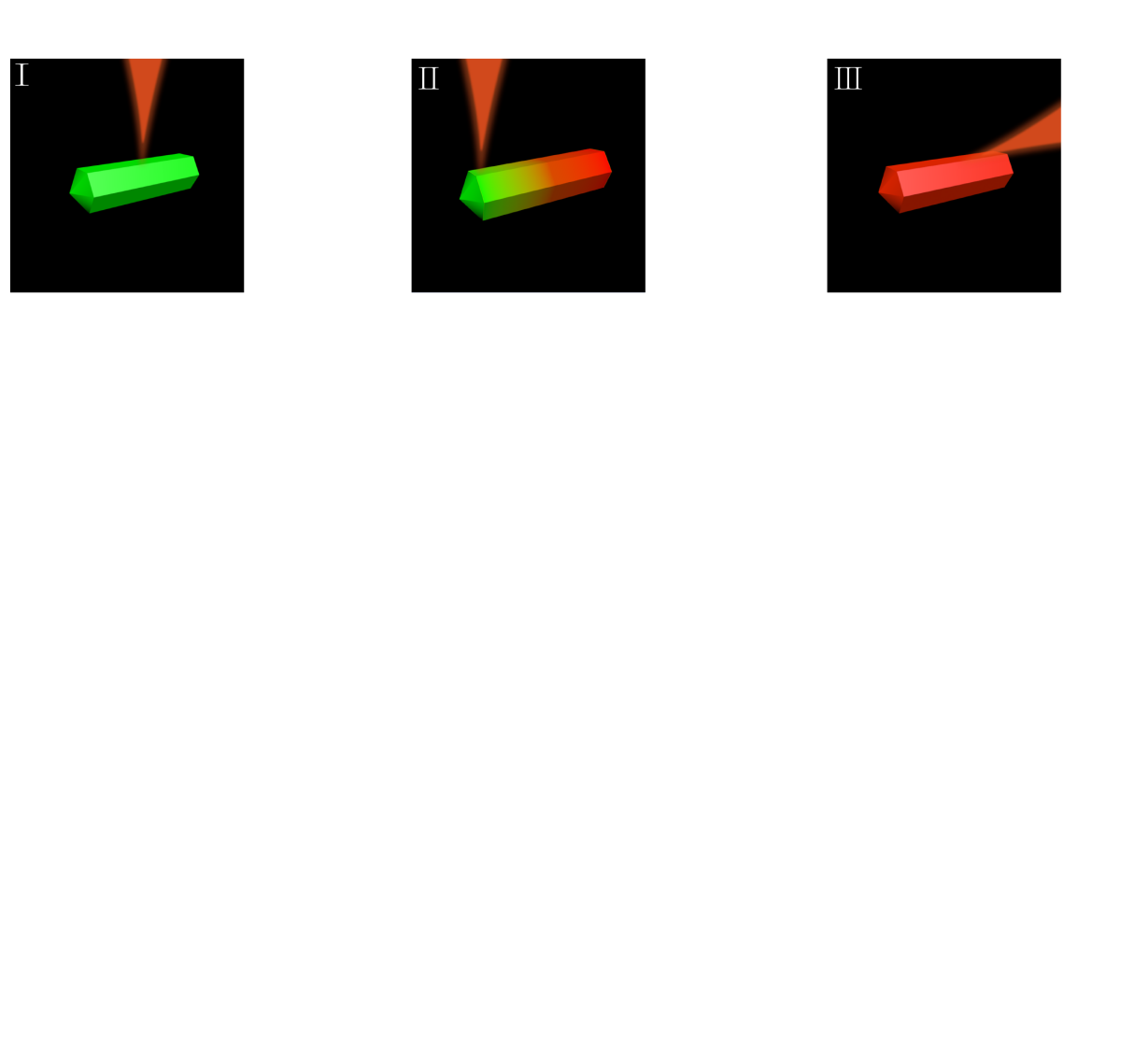


Figure S12. UC luminescence schematic structure excited at the center (Ⅰ) end (Ⅱ) and (Ⅲ) side edge of the microrod in the optical heater.

**Acknowledgements**

This work was supported by the National Key R&D Program of China (Grant No. 2020YFA0211300), the National Natural Science Foundation of China (Nos. 92050112, 92150110, 12074237, 61975101, 12004233 and 12104366), and the Fundamental Research Funds for Central Universities (GK202103010 and GK202103018, 2019TS119) and the Program of State Key Laboratory of Quantum Optics and Quantum Optics Devices (No. KF202003).

**References:**

[1] C. Zhang, J. Lu, N. Jin, L. Dong, Z. Fu, Z. Zhang, H. Zheng, “Plasmon-Driven Rapid In Situ Formation of Luminescence Single Crystal Nanoparticle,” Small, vol.15, no. 34, pp. 1901286.1-1901286.7, 2019.

[2] X. Li, D. Xiao and Z. Zhang,“Landu damping of Quantum plasmons in metal Nanostructures”New J Phys, vol.15, no. 2, pp.023011, 2013.

[3] K. Watanabe, D. Menzel, N. Nilius, et al.,“Photochemistry on Metal Nanoparticles” Chem Rev, vol.106, no. 10, pp.4301-4320, 2006.

[4] G. Baffou, R. Quidant,“Thermo-plasmonics: using metallic nanostructures as nano-sources of heat,”Laser Photonics Rev, vol. 7, no. 2, pp. 171-187, 2013.
